# Lymphomas of the Breast After Postmastectomy Implant-Based Breast Reconstruction

**DOI:** 10.1001/jamanetworkopen.2025.25820

**Published:** 2025-08-07

**Authors:** Connor J. Kinslow, Dylan K. Kim, Lauren S. Lowe, Simon K. Cheng, James B. Yu, Lisa A. Kachnic, Christine H. Rohde, David P. Horowitz, Alfred I. Neugut

**Affiliations:** 1 Department of Radiation Oncology, Columbia University Vagelos College of Physicians and Surgeons and NewYork-Presbyterian, New York; 2 Herbert Irving Comprehensive Cancer Center, Columbia University Vagelos College of Physicians and Surgeons and NewYork-Presbyterian, New York; 3 Division of Plastic and Reconstructive Surgery, Department of Surgery, Columbia University Vagelos College of Physicians and Surgeons, New York, New York; 4 Department of Radiation Oncology, James J. Peters Veterans Affairs Medical Center, Bronx, New York; 5 Smilow Cancer Center at St. Francis Hospital, Hartford, Connecticut; 6 Cancer Outcomes, Public Policy, and Effectiveness Research (COPPER) Center at Yale, New Haven, Connecticut; 7 Department of Medicine, Columbia University Vagelos College of Physicians and Surgeons and NewYork-Presbyterian, and Department of Epidemiology, Mailman School of Public Health, Columbia University, New York, New York

## Abstract

This cohort study evaluates the incidence rate of lymphomas of the breast after implant-based breast reconstruction after cancer-directed mastectomy.

## Introduction

We previously described^1^ an increase in the incidence of anaplastic large-cell lymphomas (ALCL) in association with breast implants; these adverse events have resulted in a black box warning by the US Food and Drug Administration (FDA) on all breast implants.^2^ In 2022, the FDA issued a public safety communication expanding these observations to include various non-Hodgkin lymphomas (NHL).^3^ These observation rates were limited to case reports, and there are no epidemiological studies we know of that link implants to lymphomas aside from ALCL. Here, we report the risk of lymphomas of the breast in women who underwent implant-based breast reconstruction after cancer-directed mastectomy.

## Methods

We identified women who underwent postmastectomy implant-based reconstruction for any breast tumor from January 1, 2000, to December 31, 2020, using the Surveillance, Epidemiology, and End Results (SEER) 17 database, excluding women with less than 12 months of follow-up. Participants were followed up for the occurrence of pathologically confirmed lymphomas until death, loss to follow-up, or the end of study, with a latency exclusion period of 2 months from the primary breast cancer diagnosis. Multiple primary-standardized incidence ratios (SIRs) were used to compare the number of observed vs expected cases based on incidence rates derived from the US female population, adjusted for age, race and ethnicity, and year of diagnosis.^4,5^ Statistical analyses used SEER*Stat 8.3.9 (National Cancer Institute). This study was exempt from review at Columbia University based on institutional policy for nonhuman participants research. This study followed the Strengthening the Reporting of Observational Studies in Epidemiology (STROBE) reporting guideline.

## Results

We identified 61 043 women (median [IQR] age, 51 [44-60] years; 219 [0.4%] American Indian or Alaska Native; 4565 [7.5%] Asian or Pacific Islander; 4941 [8.1%] Black; Hispanic, 6227 [10.2%]; 44 947 [73.6%] White; 144 [0.2%] unknown) with a median (IQR) follow-up of 86 (49-133) months from the incident primary breast cancer diagnosis. There were subsequently 15 NHLs of the breast (SIR, 5.03; 95% CI, 2.82-8.30), of which 7 were ALCL (SIR, 41.6; 95% CI, 16.7-85.8) and 8 were other histologies (SIR, 2.84; 95% CI, 1.23-5.60) diagnosed over 478 864 person-years (Table). This included 5 diffuse large B-cell lymphomas (SIR, 5.26; 95% CI, 1.71-12.3), 2 small lymphocytic lymphomas (SIR, 16.7; 95% CI, 2.02-60.2), and 1 peripheral T-cell lymphoma, not otherwise specified (SIR, 11.8; 95% CI, 0.30-65.7). Five were diagnosed in the breast contralateral to the primary breast cancer. Zero and 5 cases reported radiotherapy and chemotherapy, respectively, during their breast cancer treatment. The median (IQR) time from exposure to event was 83 (46-115) and 82.5 (41-144) months for breast ALCL and other NHL, respectively, with an excess risk of 14.3 and 10.8 per 1 000 000 persons per year. The excess risks of diffuse large B-cell lymphoma, small lymphocytic lymphoma, and peripheral T-cell lymphoma, not otherwise specified, were 8.5, 3.9, and 1.9 cases per 1 000 000 persons per year, respectively. We did not observe an increased risk of NHL outside the breast or Hodgkin lymphoma of the breast. The risk of NHL of the breast was not increased in women who received mastectomy without immediate implant-based reconstruction (SIR, 1.31; 95% CI, 0.91-1.83) or lumpectomy with or without radiotherapy (SIR, 1.11; 95% CI, 0.78-1.52).

**Table.** Relative and Absolute Risk of Lymphomas After Implant-Based Breast Reconstruction

Histology	Cases observed	Observed incidence^a^	Expected incidence^a^	Excess risk (95% CI)^a^	SIR (95% CI)
All lymphomas, breast	15	31.3	6.3	25.1 (11.34 to 45.74)	5.01 (2.80 to 8.26)
NHL/L-NOS, breast	15	31.3	6.2	25.1 (11.28 to 45.26)	5.03 (2.82 to 8.30)
ALCL, breast	7	14.6	0.4	14.3 (6.28 to 33.92)	41.6 (16.7 to 85.8)
NHL/L-NOS excluding ALCL, breast	8	16.7	5.9	10.8 (1.36 to 27.14)	2.84 (1.23 to 5.60)
DLBCL	5	10.4	2.0	8.5 (1.42 to 22.6)	5.26 (1.71 to 12.3)
Small lymphocytic lymphoma	2	4.2	0.3	3.9 (0.31 to 17.76)	16.7 (2.02 to 60.2)
T-NOS	1	2.1	0.2	1.9 (−0.14 to 12.94)	11.8 (0.30 to 65.7)
Hodgkin lymphomas, breast	0	0.0	0.0	−0.05 (0 to 0)	0.00 (0.00 to 168.6)
All lymphomas, nonbreast	247	516	541	25.1 (11.34 to 45.74)	0.96 (0.84 to 1.08)

Abbreviations: ALCL, anaplastic large cell lymphoma; DLBCL, diffuse large B-cell lymphoma; L-NOS, lymphoma, not otherwise specified; NHL, non-Hodgkin lymphoma; SIR, standardized incidence ratio; T-NOS, peripheral T-cell lymphoma, not otherwise specified.

^a^ Incidence and excess risk are displayed per million persons per year.

## Discussion

This cohort study reports a novel epidemiological association of breast implants with both B- and T-cell NHL; this increased risk was observed for several histologies, including diffuse large B-cell lymphoma, small lymphocytic lymphoma, and peripheral T-cell lymphoma, not otherwise specified, in addition to ALCL.

Implant-associated ALCL has been attributed to chronic inflammation, which facilitates lymphoproliferation and malignant transformation within a hypoxic tumor microenvironment.^6^ Similar causes may contribute to the pathogenesis of other NHLs, including B-cell lymphomas.

It is important to appreciate that the absolute risk of lymphoma is extremely low and similar for ALCL and the other NHL histologies. We and others have not identified an elevated risk of breast squamous cell carcinoma, another implant-associated malignant neoplasm identified by the FDA, following implant-based breast reconstruction.^3^ The FDA is aware of fewer than 30 cases of non-ALCL lymphomas in breast implant capsules vs over 1300 cases of ALCL.^3^ Continued surveillance of breast implant–associated malignanct neoplasms is warranted by government and regulatory agencies.

Limitations of the current study include our inability to evaluate women who underwent cosmetic implantation or noncancer-directed surgical procedures in the contralateral breast. Future studies should further investigate the pathophysiology, presentation, patient- and implant-specific risk factors (including implant type and manufacturer), and treatment of these malignant neoplasms.

## Notes

Supplement.
Data Sharing Statement
