# Supplementary material for: Lymphomas of the Breast After Postmastectomy Implant-Based Breast Reconstruction
Source: JAMA Netw Open. 2025 Aug 7;8(8):e2525820. doi: 10.1001/jamanetworkopen.2025.25820 (PMC12332618; doi:10.1001/jamanetworkopen.2025.25820)
Supplement: Supplement. — Data Sharing Statement [file jamanetwopen-e2525820-s001.pdf]

Data Sharing Statement

Kinslow. Lymphomas of the Breast After Postmastectomy Implant-Based Breast Reconstruction. *JAMA Netw Open*. Published August 05, 2025.
doi:10.1001/jamanetworkopen.2025.25820

Data
Data available: No

Additional Information
Explanation for why data not available: Available through request to the NCI
